# Clarifying the interaction types in two-person neuroscience research

**DOI:** 10.3389/fnhum.2014.00276

**Published:** 2014-04-30

**Authors:** Tao Liu, Matthew Pelowski

**Affiliations:** ^1^Department of Psychology, Sun Yat-Sen UniversityGuangzhou, China; ^2^Department of Psychology, University of CopenhagenCopenhagen, Denmark

**Keywords:** social neuroscience, hyperscanning, inter-brain synchronization, interaction type, cooperation, competition

Human brains and behaviors are shaped, and normally function, in continuous interaction with other humans (Hari and Kujala, 2009). However, because of the methodological difficulties related to the complex dynamics of interaction situations, neural mechanisms underlying interactive behavior remain one of the most poorly understood areas of neuroscience (Hari et al., 2013).

Previous neuroscience research has argued that social processes enabling us to interact with others are internalized and hence can be understood by investigating individual brains (see Konvalinka and Roepstorff, 2012). Based on this assumption, neuroscience studies have mainly examined social cognition from a perception perspective by presenting single participants with socially-relevant pictures or videos (Lieberman, 2007). Although this has led to identification of a set of regions composing our “social brain,” such as amygdala, orbital frontal cortex, medial prefrontal cortex, and mirror neuron system (Frith, 2007), little is still known about how these brain regions function and transfer information between brains in dynamic, real-time interactions. Especially, more recent ideas have proposed that social cognition may be fundamentally different when an individual does engage in an interaction, rather than when one just observes the situation itself (Schilbach, 2010).

## Toward a two-person neuroscience

To address this issue, a natural approach is to study the brains of two interacting people in real mutual interactions, rather than only examining single individuals in pseudo social contexts (Hasson et al., 2012; Pfeiffer et al., 2013). “Hyperscanning” is a technique that provides the possibility for simultaneous acquisition of the cerebral data from two or more participants (Montague et al., 2002). Using different hyperscanning approaches such as dual fMRI and EEG/NIRS recordings (Liu and Pelowski, in press), a few studies have examined inter-brain relationship between two participants during various interactive tasks: economic games assessing *social decision-making* (King-Casas et al., 2005; Astolfi et al., 2011); communication considering *transfer of information across brains* (Jiang et al., 2012); music playing examining *action/emotion synchrony* (Babiloni et al., 2011, 2012; Sänger et al., 2012); key-press and finger-movement tasks (Tognoli et al., 2007; Cui et al., 2012; Holper et al., 2012; Naeem et al., 2012; Yun et al., 2012) for *body-movement synchrony*; and mutual gaze task for *shared attention* (Saito et al., 2010).

These studies have shown intriguing patterns of correlation—i.e., “inter-brain synchronization”—between activations of two participants' brains, either at the same moments [e.g., in the case of Yun et al. (2012), the frontoparietal network simultaneously activated when participants coordinated movements of fingertips] or in turn-based time delay [Jiang et al. (2012) showed left inferior frontal gyrus (IFG) synchronization in face-to-face communication]. These findings demonstrate promise of hyperscanning for understanding the neural mechanisms of exchanging and sharing information between two brains underlying interpersonal interaction, which cannot be detected when only examining single brains (Babiloni and Astolfi, 2012; Chatel-Goldman et al., 2013). For this reason, a growing number of researchers have called for new investigation of “social interaction and its relationship to social cognitive abilities in more ecologically valid ways” (Schilbach et al., 2013), emphasizing the importance of a “second-person neuroscience” or a “two-person neuroscience” (Hari and Kujala, 2009).

## What does synchronization mean?: a need for paradigm classification in hyperscanning research

Although recent hyperscanning studies have revealed intriguing synchronization, it remains difficult to explain exactly *how* and *why* inter-brain synchronization occurs within such research (Konvalinka and Roepstorff, 2012). As noted by Liu and Pelowski in a recent review, present research paradigms raise some important questions: In order to act synchronously, do interacting agents need to behave the same way at the same time leading to inter-brain synchronization? Alternatively, might synchronization also entail any number of other lawful relations between agents (not the same time/behavior)? Specifically, it is quite possible that by doing the same task in the same environment, two brains activate in similar ways, having little to do with the presence of the other human.

One of the important reasons for this limitation is lack of clear understanding or classification of the interaction tasks used in previous hyperscanning studies. Namely, it may be the paradigm, as well as paradigm differences, creating the present results or major differences in previous research. This paper therefore will review existing tasks with an eye toward impact on hyperscanning results and considering possible parallels or differences. We will also conclude with a suggestion for future study, that might aid in clarifying the structure of interactions and provide reference for hyperscanning research.

## Interaction structures: a review of hyperscanning tasks

**Figure 1:**
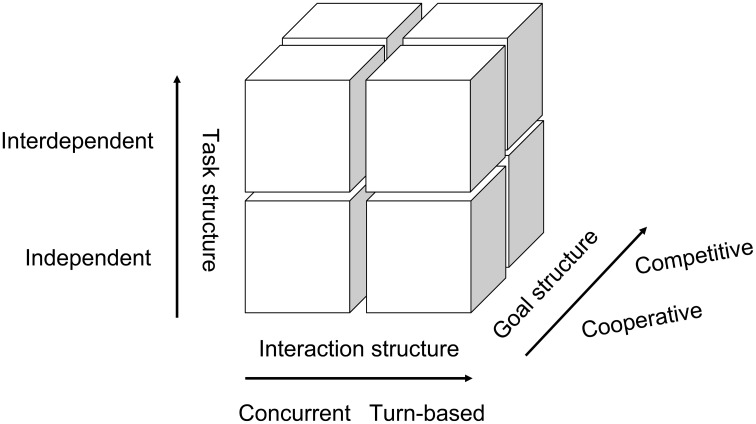
**(A)** Potential interaction structures in hyperscanning research.

Figure 1 illustrates the structure of interaction tasks proposed by this paper. Human behaviors are normally affected by three factors when interacting with other people in a social context: (1) interaction structure, (2) goal structure, and (3) task structure. The x-axis represents the interaction structure. Interaction is defined as “individual's simultaneous or sequential actions that affect the immediate and future outcomes of the other individuals involved in the situation” (Johnson and Johnson, 2005). Thus, Liu and Pelowski (in press) have categorized social interaction into two structures: concurrent interaction requiring body-movement synchrony between two people (i.e., the same behavior with or without time-delay, such as pair Olympic diving) and turn-based interaction that relies primarily on mind-set synchrony—i.e., holding representations of one's own intention and that of others simultaneously for complementary or contrary behavior, such as in a game of chess.

The y-axis indicates the goal structure. Individuals may either facilitate the goal achievement of others (cooperation) or obstruct others (competition) (Deutsch, 1949).

The z-axis represents task structure. There are two main interactive tasks, depending on whether the task requires mutual dependence between participants (Johnson et al., 1981; Tauer and Harackiewicz, 2004). During *interdependent* tasks such as tennis, both individual behavior and outcome are affected by each other. In contrast, during *independent* tasks, such as a race, individuals complete the task independently, while final outcome (winning or losing) is determined by the other.

Social psychology literature has consistently demonstrated that different types of interaction may involve different cognitive processes and behaviors (Johnson et al., 1981; Johnson and Johnson, 1989). Therefore, in order to fully understand neural mechanisms underlying human behavior, it is important to clearly separate and examine each type of interaction itself. According to the proposed classification system (Figure 1), there are generally eight interaction types, which we will now consider. We will first discuss studies in concurrent interactions (four types) and then review turn-based (four types).

## Concurrent interactions

First, looking to current research, previous hyperscanning studies have mainly examined concurrent *interdependent* cooperation tasks. This has involved key-press (Funane et al., 2011; Cui et al., 2012), finger-movement tasks (Tognoli et al., 2007; Holper et al., 2012; Naeem et al., 2012; Yun et al., 2012) and music-playing tasks (Lindenberger et al., 2009; Sänger et al., 2012). Results demonstrate that concurrent interdependent cooperation requires prediction of other's behavior for synchronized body-movements, and has been associated with inter-brain synchronization in right frontoparietal regions.

Regarding concurrent *independent* cooperation, Dommer et al. (2012) have simultaneously measured pairs of participants' activation in a dual n-back task and revealed inter-brain synchronization in prefrontal cortex (PFC).

On the other hand, Cui et al. (2012) have also studied concurrent independent *competition*, asking pairs of participants to press two keys as fast as possible to beat their partner, and demonstrated no inter-brain synchronization. This result suggests that individuals may complete the concurrent independent competition without understanding of the other's actions/mind. Therefore, resulting synchronization in paradigms relating to cooperation, but not competition, was argued to be one of the clearest indications of interplay or sharing of information between brains.

At the same time, returning to the structure posed by our figure, careful discussion is needed for the interpretation of these synchronization results in concurrent cooperation/competition. E.g., synchronization in the *concurrent* tasks, whether independent/interdependent, completion or cooperation, may result from the functional similarity between two participants' task/environment, rather than the specific neural mechanism underlying interactive behavior (Sänger et al., 2012; Liu and Pelowski, in press). That is, future research needs to look at these other two factors, factoring out concurrent behaviors, in order to better assess veracity of brain synchronization. On the other hand, lack of synchronization in the case of Cui et al., which one might be tempted to compare to findings of synchronization in the interdependent tasks, may in fact be result of task *independence*, rather than a factor of lack of communication between brains. This raises the need for future research to be mindful whether comparisons can be made between Figure paradigms.

No research has investigated concurrent interdependent *competition*, marking a clear avenue for future work.

## Turn-based interactions

In turn-based behaviors, previous hyperscanning studies have examined inter-brain relationship between two participants using tasks such as communication (Jiang et al., 2012) and economic games (Astolfi et al., 2011). Results revealed that inter-brain synchronization in the IFG is associated with successful face-to-face communication (i.e., turn-based *interdependent cooperation*), since there is no inter-brain synchronization between a speaker and a listener who cannot understand the language of the other (Stephens et al., 2010). In contrast, economic games, e.g., the Prisoner's Dilemma, focusing primarily on *independent* cooperative decision lead to inter-brain synchronization in the PFC and anterior cingulated cortex (ACC). This has been argued to play a role in reading each others' mind (Vogeley et al., 2001).

To examine neural mechanisms underlying turn-based *interdependent* behavior, Liu et al. (2013, under review) have measured activation of paired participants using a computerized turn-taking game designed by Decety et al. (2004). One member of a pair of participants was assigned to a builder role, with the task of copying a disk-pattern on a monitor, while the partner's task was to either aid the builder in his goal (cooperation condition) or obstruct (competition). Results showed inter-brain synchronization in the IFG during cooperation, and further demonstrated synchronization in the IFG and the inferior parietal lobule (IPL) during competition.

Astolfi et al. (2010) made similar study of cooperation, simultaneously measuring four participants' brains in a Bridge-like card game. Participants were paired into two teams and played the card game in a turn-taking style, cooperating to beat the other team. Results revealed inter-brain synchronization between ACC activity of the second player of a team and the PFC activity of the other player of the same team. According to the authors, this result was consistent with previous argument that the PFC is associated with evaluation of uncertainty and risk, while ACC is associated with conflict monitoring and intention understanding.

However, here questions need to be raised regarding ability to compare across interaction types. In turn this specific type, and especially the finding of Astolfi et al. also raise need for further control to be sure of which category research should be placed within. From a hyperscanning perspective, one possible interpretation of the PFC-ACC synchronization is that the same team members may play the card game as a *turn-based independent cooperation* task such as in the Prisoner's Dilemma. It is noteworthy that every player cooperated with their in-team partner and competed with their out-team opponents as well. To examine whether or not the results of Astolfi et al. (2010) involve different components, further study is needed to investigate the inter-brain relationship of turn-based independent cooperation and competition separately.

Again, it is also intriguing to note that no study has investigated inter-brain relationship during turn-based *independent competition* such as a game of darts.

## Conclusion

To clearly understand the inter-brain mechanisms underlying human interactive behavior, it is important to clarify the interaction types in the initial two-person neuroscience studies. Specifically, clear separation of interaction types is important to the interpretation and understanding of inter-brain synchronization revealed in various experimental tasks. Concurrent interdependent cooperation may be associated with inter-brain synchronization in the mirror neuron system of two participants, while concurrent independent cooperation may be associated with inter-brain synchronization in the PFC. In particular, Cui et al. (2012) revealed no inter-brain synchronization in the concurrent independent competition, whereas Liu et al. (2013, under review) revealed inter-brain synchronization in participants' mirror neuron system in the turn-based interdependent competition. These results imply that different types of interaction may involve distinctive neural mechanisms, requiring careful separation and interpretation according to the proposed classification system in the future studies.

### Conflict of interest statement

The authors declare that the research was conducted in the absence of any commercial or financial relationships that could be construed as a potential conflict of interest.
